# Positive adult experiences as turning points for better adult mental health after childhood adversity

**DOI:** 10.3389/fpubh.2023.1223953

**Published:** 2023-08-03

**Authors:** AliceAnn Crandall, Brianna M. Magnusson, Melissa J. Barlow, Hannah Randall, Abigail L. Policky, Carl L. Hanson

**Affiliations:** Department of Public Health, Brigham Young University, Provo, UT, United States

**Keywords:** positive adult experiences, positive childhood experiences, adverse childhood experiences, depression, anxiety

## Abstract

**Background:**

The purpose of this study was to examine whether positive adult experiences (PAEs) were associated with lower odds for anxiety and depression even in the presence of high adverse childhood experiences (ACEs) or low positive childhood experiences (PCEs).

**Methods:**

The sample was comprised of 435 adults (48% female), ages 18–56 years and who were living in the United States. Participants completed a survey about their childhood experiences, PAEs, and mental health. A series of multiple logistic regression models were estimated in Stata 17 to examine the aims.

**Results:**

Positive childhood experiences were associated with higher PAE scores, but ACEs did not significantly correlate with PAEs. Positive adult experiences were associated with lower odds of moderate-to-severe anxiety and depression, especially among those who had experienced high ACEs or low PCEs. Younger adults were more likely to experience a positive benefit from PAEs compared to adults 35 years and older.

**Conclusion:**

Even when ACEs were high or PCEs were low, adults with high PAEs had lower odds for moderate-to-severe anxiety and/depression. Positive adult experiences may be an opportunity to turn the tide for individuals who experienced childhood adversity and/or low levels of support or connection.

## 1. Introduction

Multiple recent studies have demonstrated that positive childhood experiences (PCEs) lead to better health in adulthood even in the presence of adverse childhood experiences (ACEs) (1–4). In particular, PCEs appear to improve mental health (2, 3), and in some cases the absence of PCEs may be linked with more mental health problems than the presence of ACEs (3). PCEs, like ACEs, are cumulative experiences that occur before age 18 years. They include healthy relationships with parents, teachers, other adults, peers, and one’s community as well as stable home routines and having a purpose in life.

Although a focus on promoting positive experiences in childhood is important for preventing trauma and represents an important direction for combatting the negative effects of ACEs, consideration should also be given to adults who did not experience a high number of PCEs to consider how best to support health and wellbeing in adulthood. An important next research step is examining whether positive experiences that occur in adulthood (positive adult experiences, or PAEs) may also lead to improved mental health in adulthood, even when ACEs were high and/or PCEs were low. PAEs include positive relational health in a variety of relationships (e.g., family, friends, neighbors, community) as well as other positive factors such as healthy routines, positive self-care, engaging in meaningful work or other activities, and having beliefs that give them comfort.

### 1.1. Developing resilience in adulthood after childhood trauma

Evidence indicates that there are circumstances where one may experience stress or adversity earlier in life which can strengthen resilience later in life, which is called the “steeling effect” (5). For example, some adults who experienced great hardship due to the Great Depression during their childhood were more able to cope with difficult situations in adulthood (5). However, given the volume of research showing that childhood adversity can be very harmful to adult physical and mental health (6, 7) it cannot be assumed that cumulative childhood trauma will lead to a steeling effect in most adults. Instead, it is important to consider what beneficial experiences during adulthood serve as “turning points” (5) that help to combat prior traumatic life experiences and improve health. Turning points exert an independent positive impact on wellbeing even when there was prior adversity such as ACEs. One example of a turning point is a supportive marriage, which can give an adult a secure and safe relationship to help them through future hardships in adulthood (5, 8). Beyond marriage, other healthy relationships including feeling a sense of belonging in the community and having social supports contribute to higher levels of both physical and mental health among adults (9). Greater social connection may foster a sense of “coherence” or meaning and purpose in life that enhances adult mental health, physiological processes, and physical health (10). Further, there is a clear connection between higher levels of community belonging and better health outcomes among adults from both urban and rural areas as well as diverse socioeconomic backgrounds (11).

Beyond healthy relationships and belonging, there are other potential turning points. Personal religious practices founded on genuine personal beliefs beyond the actions of religious practice alone decrease mortality and can improve mental health (12, 13). Involvement in positive health-promoting self-care behaviors is related to a reduced likelihood of developing symptoms of anxiety and depression (14). Mental health can also be related to behavior changes. For example, at the start of the COVID-19 pandemic, those who had been physically active and less sedentary maintained good mental health while those who increased screen time and lessened physical activity experienced lower levels of mental health (14).

### 1.2. Theoretical perspectives

Several theoretical perspectives help to explain the influences of early childhood experiences on health outcomes and include the compensatory model of resilience, cumulative experiences models, and the stress buffering model.

#### 1.2.1. Compensatory model of resilience

The compensatory model of resilience posits that positive factors have a direct, independent effect on an outcome even in the presence of a risk factor (15). These positive factors may neutralize the effect of risk factors on the outcome. In statistical analyses, compensatory factors and risk factors are both included in a regression model as independent variables. As it relates to the current study, PAEs, which cumulatively serve as turning points, are the positive factors that exert a direct effect on adult mental health even in the presence of ACEs. They may help to neutralize the negative impact of potentially traumatic events in childhood on adult wellbeing.

#### 1.2.2. Cumulative experiences models

Cumulative experiences models help to explain how negative and positive life experiences impact health and are rooted in both the cumulative stress model and the cumulative resiliency model. The cumulative stress model (or allostatic load model) proposes that stressful experiences, accumulate across the lifespan to influence adult health outcomes (16). Studies have demonstrated that the accumulation of life stressors, referred to as allostatic load, leads to the dysregulation of the hypothalamic–pituitary–adrenal (HPA) axis leading to acute and chronic disease (17–20). Evidence also exists that link ACEs to elevated higher allostatic load and adverse health outcomes in adulthood (21).

Similarly, the cumulative resiliency model purports that positive experiences such as PCEs accumulate across the lifespan to influence health outcomes. In research relating to PCEs, it is the accumulation of PCEs and not just a single experience that is predictive of improved lifelong health (1–3). It is probable that in the ‘turning point’ research cited above, the single event in adulthood was founded on the accumulation of other positive events. For example, a supportive marriage does not occur in a vacuum, rather accumulated experiences assist in developing skills that enhance an individual’s ability to create and maintain healthy relationships. Thus, similar to PCEs, the more positive experiences that accumulate in adulthood, the more likely PAEs can serve as ‘turning points’ even when adversity in childhood was high.

#### 1.2.3. Stress buffering model

The stress buffering model purports that having supportive relationships protects individuals from the harmful effects of stress on health outcomes (22, 23). The mechanism through which stress is buffered can be explained in the transactional model of stress and coping (24) where social support may impact appraisal response (25). As prior research has indicated that ACEs lead to worse physical and mental wellbeing in childhood while PCEs lead to better physical and mental wellbeing regardless of ACE score (1–3), it is likely that ACEs are associated with fewer PAEs and PCEs with more PAEs. As such, PAEs may mediate the relationship between childhood experiences and adult wellbeing. PAEs may also serve as moderating (buffering) variables between childhood experiences and adult health, with the accumulation of positive experiences buffering the relationship between early adversity and later wellbeing.

Few studies have explored the buffering influence of positive adult experiences (PAEs) in the pathways between ACEs/PCEs and adult mental health outcomes. There is some evidence that resilience resources such as a sense of community and health habits help to ameliorate (moderate) the risk of ACEs that lead to adult mental health problems (26). In another study, positive adult life events as measured by a substantial increase in income or improvement in financial situation, positive change in employment, development of a new important friendship, or receiving an award, honor or recognition were not found to buffer the effects of ACEs on adult allostatic load, or cumulative biological risk for adverse health outcomes (27).

### 1.3. Gaps in the literature

Although there is literature on the positive benefits of specific events in adulthood, to our knowledge there is no literature examining the accumulation of positive experiences in adulthood and its direct, mediating, and moderating effects on mental health, after accounting for both ACEs and PCEs. Another gap in the current literature is understanding the relationship of childhood adversity and PAEs on adult health in younger vs. older adults in retrospectively reported childhood experiences. Age when trauma was experienced in childhood can impact how the experience affects individuals in adulthood (28). Further, it is possible that over time adults forget childhood adversities that still impact them (29). As such, the effects of PAEs on adult health may diminish over time as childhood adversities become more distal or diluted with the accumulation of more proximal events. Retrospective and prospective reports of childhood adversity have shown moderate agreement (29). Some childhood adversity, such as sexual abuse, is more likely to be underreported in prospective studies, while some ACEs are forgotten over time and thus underreported in retrospective studies (29). Beyond the effects of reporting childhood experiences, the effects of PAEs on health may vary based on developmental trajectories in adulthood. For example, younger adults are going through many important life transitions (e.g., marriage, education, employment, and housing relocation) that may be more influenced by the PAEs they have. Furthermore, mental illness is more prevalent in younger adults vs. middle and older adults (30), and thus PAEs might be more important to young adult health when mental illness is the outcome.

### 1.4. Current study

In the current study, PAEs are intended to measure a breadth of positive experiences in adulthood that acting together can lead to better health regardless of the presence of ACEs or absence of high PCEs. Like ACEs and PCEs, PAEs measure cumulative advantage but do not measure what experiences in adulthood are most important to better mental health. Instead, they provide a snapshot of the breadth of positive experiences that an adult may experience. PAEs include several items similar to those in common to PCE instruments [e.g., the Positive Childhood Experiences and the Benevolent Childhood Experiences scales (1, 2)] and some items that may be particularly important to adult wellbeing and resilience. Building off of the prior research demonstrating aspects of adult life that lead to better outcomes, PAEs examine adult relationships with family, friends, and community (e.g., having people to talk to about feelings or feeling supported in one’s relationships) as well as adult life stability and meaning (e.g., opportunities to engage in meaningful daily activities, having daily routines, and having beliefs that provide comfort).

The study had four primary aims. Aim 1 was to examine the effects of childhood experiences on PAEs. We hypothesized that those with higher ACEs would have lower PAEs and those with higher PCEs would also have more positive experiences in adulthood. In Aim 2, we examined the relationship between childhood experiences and PAEs on adult depression and anxiety. We hypothesized that PCEs and PAEs would both be linked with lower rates of anxiety and depressive symptoms and that ACEs would be associated with modestly higher rates of anxiety and depression. The purpose of aim 3 was to explore whether PAEs led to better adult mental health even when PCEs were below mean scores or ACEs were high (e.g., ≥4 ACEs). We hypothesized that PAEs could be turning points (5) that led to better adult mental health even in the presence of lower PCEs or high ACEs. Finally, for aim 4 we examined if the effects of PAEs on adult mental health varied among young adults compared to middle-aged adults. We hypothesized that PAEs would be beneficial in both age groups, but have more of a positive effect among young adults.

## 2. Materials and methods

The final sample was comprised of 435 adults, ages 18–56 years who were living in the United States. Participants were recruited via Amazon Mechanical Turk (MTurk) during September 2021. To ensure that comparisons could be made in younger (e.g., 18–34 years) and middle age adults (e.g., 35–56), participants were recruited in batches by age (e.g., 18–25, 25–35, 35–45, and so forth).

MTurk is a crowdsourcing recruitment service, allowing researchers to gather a research panel of participants who meet study qualifications based on their profile information in MTurk. For the current study, eligible participants were able to view a brief description of the study and provided a link to the Qualtrics survey that contained a consent statement. Participants who agreed to participate were then able to view and complete the survey. The survey took approximately 15 min to complete. Following completion and a quality check, participants were given a $2.00 credit in their MTurk account. Approximately 10% of completed surveys were rejected due to poor quality (e.g., completed in less than half of the median time, answers demonstrated straight-lining, or reported age was outside of the age range for the batch). Compared with other survey services, MTurk participant demographics are similar and MTurk samples have good generalizability to national samples (31, 32).

### 2.1. Measures

#### 2.1.1. Mental health

Depression was measured using the 9-item Patient Health Questionnaire (PHQ-9) (33). Anxiety was measured using the 7-item Generalized Anxiety Disorder scale (GAD-7) (34). For both scales, response options were on a 4-point Likert scale ranging from *Not at all* to *Nearly every day*. Items were summed and then dichotomized, with scores of 10 or higher (coded as 1) indicating moderate-to-severe depression/anxiety and scores below 10 coded as 0 (no or mild depression/anxiety) (33, 34). The PHQ-9 has historically had strong reliability (*α =* 0.89) (33). The current study also demonstrated high reliability (*α =* 0.95). Likewise, the GAD-7 has previously reported excellent reliability (*α =* 0.92) (34), and had excellent reliability in the current sample (*α =* 0.95).

#### 2.1.2. Positive adult experiences

Although prior research has examined individual positive experiences in adulthood (e.g., social connection), to our knowledge there was not a measure of cumulative positive experiences in adulthood that was analogous to cumulative childhood measures. As such, a 15-item questionnaire was developed to measure PAEs. A team of researchers, including the authors of this paper, developed the items. Some items were adapted to fit the adult context from the Benevolent Childhood Experiences scale (PAEs items 6–8, 10–12, 14) (1) and Positive Childhood Experiences scale (PAEs items 1–4) (2) while other items were selected based on the ‘turning points’ research indicating their importance to adult wellbeing. Cognitive interviews were conducted with young and middle adult males and females to assess their interpretation of questions and their perceptions of important questions to include. Based on these interviews, additional questions were added (PAEs items 5, 9, 13, and 15). Response options were dichotomous (yes/no). The “yes” responses were summed for a cumulative PAEs score. The items, including mean scores, are in Table 1. Table 2 includes item tetrachoric correlations, which is appropriate for binary data. Further analysis indicated that the scale fits as a single factor. The measure had adequate reliability (*α* = 0.77). In factor analysis, the single eigenvalue was 6.14 and Geomin factor loadings ranged from 0.43 (In the past 2 weeks, I have talked to somebody outside of my family about my feelings) to 0.80 (I feel like I belong in my community).

**Table 1:** Percentage reporting each positive adult experience (PAE) in a sample (*N* = 435) of 18–56 year old adults in the U.S.

PAE Item	%
In the past 2 weeks, I have talked with at least one member of my family about my feelings.^a^	82.99
In the past 2 weeks, I have talked to somebody outside of my family about my feelings. ^a^	66.21
I feel that my family stands by me during difficult times. ^a^	87.59
I enjoy participating in community events and/or traditions. ^b^	71.49
I feel like I belong in my community.	71.72
I feel supported by my friends. ^b^	87.33
I have at least one close and lasting relationship where I can depend on the other person to help me make decisions about my life. ^b^	86.67
I feel safe with those who live in my home. ^b^	94.48
My residence and neighborhood are safe.	89.89
I have good neighbors. ^b^	89.66
I have beliefs that give me comfort. ^b^	88.97
I have done something fun in the past 2 weeks. ^b^	85.75
In the past 2 weeks, I have done things to take care of myself such as reading a book, getting a massage, or going for a run.	87.59
I have a predictable home routine, like a set time to exercise and a regular bedtime. ^b^	78.62
I am engaged in daily activities that are rewarding and meaningful to me, such as volunteering in the community, having a job I enjoy, or parenting.	81.84

^a^Item adapted from the Positive Childhood Experiences Scale (2). ^b^Item adapted from the Benevolent Childhood Experiences Scale (1).

**Table 2:** Correlation matrix for pairwise tetrachoric correlations between Postiive Adult Experiences Items in a sample of 18–56 years old U.S. Adults (*n* = 435).

Variables		pae1	pae2	pae3	pae4	pae5	pae6	pae7	pae8	pae9	pae10	pae11	pae12	pae13	pae14	pae15
pae1	In the past 2 weeks, I have talked with at least one member of my family about my feelings.	1.00														
pae2	In the past 2 weeks, I have talked to someone outside of my famiy about my feelings.	0.42*	1.00													
pae3	I feel that my family stands by me during difficult times.	0.52*	−0.19	1.00												
pae4	I enjoy participating in community events and/or traditions.	0.39*	0.40*	0.15	1.00											
pae5	I feel like I belong in my community.	0.46*	0.36*	0.39*	0.76*	1.00										
pae6	I feel supported by my friends.	0.39*	0.42*	0.33*	0.56*	0.63*	1.00									
pae7	I have at least one close and lasting relationship where I can depend on the other person to help me make decisions about my life.	0.33*	0.46*	0.41*	0.27*	0.45*	0.57*	1.00								
pae8	I feel safe with those who live in my home.	0.16	0.12	0.60*	0.40*	0.20	0.25	0.17	1.00							
pae9	My residence and neighborhood are safe.	0.08	0.07	0.42*	0.19	0.39*	0.14	0.38*	0.51*	1.00						
pae10	I have good neighbors.	0.24*	0.13	0.33*	0.37*	0.46*	0.43*	0.33*	0.44*	0.62*	1.00					
pae11	I have beliefs that give me comfort.	0.40*	0.32*	0.31*	0.59*	0.54*	0.45*	0.46*	0.49*	0.29*	0.33*	1.00				
pae12	I have done something fun in the past 2 weeks.	0.02	0.34*	0.24*	0.31*	0.21*	0.36*	0.60*	0.22	0.46*	0.13	0.36*	1.00			
pae13	In the past 2 weeks, I have done things to take care of myself such as reading a book, getting a massage, or going for a run.	0.28*	0.32*	0.30*	0.18	0.16	0.25*	0.47*	0.55*	0.34*	0.33*	0.39*	0.48*	1.00		
pae14	I have a predictable home routine, like a set time to exercise and a regular bedtime.	0.10	0.24*	0.36*	0.35*	0.43*	0.22*	0.39*	0.26	0.39*	0.28*	0.33*	0.35*	0.17	1.00	
pae15	I am engaged in daily activities that are rewarding and meaningful to me, such as volunteering in the community, having a job I enjoy, or parenting.	0.33*	0.11	0.49*	0.55*	0.55*	0.60*	0.45*	0.52*	0.30*	0.46*	0.67*	0.56*	0.43*	0.51*	1.00

*p < 0.05.

#### 2.1.3. Childhood experiences

A 13-item positive childhood experiences (PCEs) scale (35) was used that included 10 items from the Benevolent Childhood Experiences scale (1) and three items from the Positive Childhood Experiences scale (2). The scale had good reliability (*α* = 0.84). Sample items included “When you were growing up, during your first 18 years of life…Did you feel that your family stood by you during difficult times?” and “When you were growing up, during your first 18 years of life…Did you have at least one caregiver with whom you felt safe?” Response options were “Yes” (coded as 1) and “No” (coded as 0). A composite PCE score was created by summing the “yes” responses, with possible scores ranging from 0 to 13.

Adverse Childhood Experiences (ACEs) were measured using the 11-item ACEs module from the Centers for Disease Control and Prevention’s Behavioral Risk Factor Surveillance System Survey (Centers for Disease Control and Prevention, 2016). Like the PCE measure, response options were dichotomous (1 = yes; 0 = no), and the responses were summed for a total cumulative score of adversity ranging from 0 to 8 ACEs (substance use in the home items were combined as were sexual abuse items). Sample items included “When you were growing up, during your first 18 years of life…Did you live with anyone who was depressed, mentally ill, or suicidal?” and “When you were growing up, during your first 18 years of life… Did you live with anyone who served time or was sentenced to serve time in a prison, jail, or other correctional facility?” The ACEs measure had good internal reliability (*α* = 0.80).

#### 2.1.4. Other covariates

Gender (1 = female; 0 = male), age (in years), education (1 = Bachelor’s degree or higher; 0 = less than a Bachelor’s degree), race (1 = White; 0 = non-White), and marital status (1 = married; 0 = not married) were included as model covariates.

### 2.2. Data analysis

Data were cleaned and analyzed in Stata version 17. Multiple linear regression was used to examine the effects of childhood experiences on PAEs. To examine the relationship between childhood experiences and PAEs on adult mental health, multiple logistic regression was used, with separate models for anxiety and depression. ACEs, PCEs, and PAEs were included in these models as continuous variables. Next, a series of multiple logistic regression models were fit to examine the respective influence of ACEs and PAEs on depression and anxiety in samples with PCEs equal to or higher than the median of 12 (>11 PCEs) vs. samples with low-to-moderate (or lower than the median) PCEs (0–11 PCEs). A separate set of models were run examining high ACEs (≥ 4 ACEs) compared to low or moderate ACEs (<4 ACEs). Finally, multiple logistic regression models were run to examine differences in how childhood experiences and PAEs influenced adult depression and anxiety among younger vs. middle-aged adults by dividing the sample by those who were 18–34 years versus those who were 35 years or older. When included as independent variables in these models, ACEs, PCEs, and PAEs were included as continuous variables. All models included participant gender, age, education, race, and marital status as controls.

## 3. Results

Table 3 includes the full descriptive results of the sample. Participants were on average 37 years old, with 48% reporting their sex as female. The sample was 74% White race, 69% had at least a Bachelor’s degree, and 30% had a household income under $40,000/year. Forty percent of the sample reported moderate-to-severe depression and/or anxiety in the past 2 weeks, with 37% experiencing moderate-to-severe depressive symptoms, 31% experiencing moderate-to-severe anxiety symptoms, and 29% experiencing both moderate-to-high anxiety and depressive symptoms. Participants had on average 12.5 PAEs, 2.5 ACEs, and 10.7 PCEs, with 35% reporting four or more ACEs. Participants who had the median or higher PAEs (median PAE score = 13) were more likely to be married, report their race as White/Caucasian, have a Bachelor’s degree, and have a higher PCEs score; they were less likely to have an income below $40,000/year, to experience moderate-to-severe anxiety or depressive symptoms, and reported fewer ACEs.

**Table 3:** Descriptive statistics, *N* = 435.

Sample characteristic	Total sample	At or above median (≥13)	Below median (<13)
	(*n* = 435)	(*n* = 203)	(*n* = 232)
	%	%	%
Age [M (SD)]	36.59 (9.40)	36.83 (9.21)	36.37 (9.58)
Female	47.59	50.25	45.26
Married	56.32	67.00*	46.98*
White	73.79	78.33*	69.83*
Household income < $40,000/Year	29.89	21.67*	37.07*
Bachelor’s degree or Higher	68.51	76.85*	61.21*
Currently employed	88.74	91.63	86.21
Moderate-to-severe depression	37.24	28.57*	44.83*
Moderate-to-severe anxiety	31.03	24.14*	37.07*
≥4 ACES^a^	34.94	26.60*	42.24*
>11 PCEs^b^	53.33	80.79*	29.31*
	*M (SD)*	*M (SD)*	*M (SD)*
ACEs Score (R: 0–8)^a^	2.53 (2.29)	2.02 (2.23)*	2.97 (2.25)*
PCEs Score (R: 0–13)^b^	10.66 (2.86)	12.05 (1.91)*	9.45 (2.99)*
PAEs Score (R: 0–15)^c^	12.51 (2.67)	14.66 (0.47)*	10.62 (2.35)*

^a^Adverse childhood experiences (ACEs). ^b^Positive childhood experiences (PCEs). ^c^Positive adult experiences (PAEs). *Indicates significant difference between groups at *p* < 0.05.

### 3.1. Aim 1: childhood experiences and PAEs

Table 4 includes the results for aim 1. ACEs were not associated with PAEs, but each additional PCE was associated with an increase in the PAE score of 0.53 (*p* < 0.001). Females and those who were married also reported higher PAEs.

**Table 4:** Adjusted odds ratios for childhood and adult experiences and mental health, *N* = 435.

	PAEs score^a^	Depression (OR)	Anxiety (OR)
PAEs score	--	0.73***	0.84**
ACEs score	−0.07	1.69***	1.66***
PCEs score	0.53***	1.15*	1.06
Female	0.52*	1.36	1.47
Age	−0.00	0.96**	0.97
Bachelor’s Degree	0.12	2.20**	2.16*
White	0.29	1.72	1.68
Married	0.92***	1.94*	1.23

*p < 0.05; **p < 0.01; ***p < 0.001. ^a^Results in this column are betas from multiple linear regression. PAEs, positive adult experiences; ACEs, adverse childhood experiences; PCEs, positive childhood experiences. PAEs, ACEs, and PCEs were included in the model as continuous variables.

### 3.2. Aim 2: childhood experiences, PAEs, and mental health

PAEs were associated with lower odds of depression (0.73, *p* < 0.001) and anxiety (0.84; *p* < 0.01) while ACEs were associated with higher odds of depression (1.69, *p* < 0.001) and anxiety (1.66, *p* < 0.001). PCEs were not associated with anxiety and were modestly associated with higher odds of depression (1.15, *p* < 0.05) (see Table 4 for full results).

Because the data were cross-sectional, we did not examine the indirect effects of childhood experiences and adult mental health with PAEs as a mediator in our primary analyses. However, in a sensitivity analysis, we used a path model in Mplus Version 7 to examine indirect effects. Using 5,000 bootstraps to ensure robust standard errors, results indicated that PAEs mediated the relationship between PCEs and moderate-to-severe depression (standardized *z-score* = −4.94, *p* < 0.001) and between PCEs and moderate-to-severe anxiety (standardized *z-score* = −3.03, *p* < 0.01). The indirect effects of PAEs for ACEs and later adult mental health were not significant. See Table 5 for full results.

**Table 5:** Indirect effects of childhood experiences and adult mental health, with PAEs as the mediator.

Indirect pathway	*b*	Standardized *Z*	*p*-value
ACEs - > PAEs - > Depression	0.02	1.40	0.160
PCEs - > PAEs - > Depression	−0.20	−4.94	<0.001
ACEs - > PAEs - > Anxiety	0.01	1.29	0.196
PCEs - > PAEs - > Anxiety	−0.12	−3.03	0.002

Model uses 5,000 bootstraps to ensure robust standard errors. PAEs, positive adult experiences; ACEs, adverse childhood experiences; PCEs, positive childhood experiences. PAEs, ACEs, and PCEs were included in the model as continuous variables.

### 3.3. Aim 3: the association of PAEs and adult mental health among participants with low PCEs or high ACEs

PAEs were associated with 21% lower odds of moderate-to-severe depression among those with low-to-moderate PCEs (see Table 6). However, the relationship between PAEs and depression was not significant among those with high PCEs. Similarly, PAEs were associated with 15% lower odds of moderate-to-severe anxiety among those with low-to-moderate PCEs but were not associated with anxiety with those with high PCEs. ACEs were associated with higher odds of depression and anxiety in both the high PCE sample and the low-to-moderate PCE sample.

**Table 6:** Adjusted odds ratios of PAEs on mental health in subsamples of high vs. low/moderate PCEs.

	Depression		Anxiety	
	At or above median PCEs (≥12) (*n* = 232)	Below median PCEs (<12) (*n* = 203)	At or above median PCEs (≥12) (*n* = 232)	Below median PCEs (<12) (*n* = 203)
PAEs	0.79	0.79***	1.01	0.85*
ACEs	1.98***	1.48***	1.93***	1.44***
Female	2.99**	0.77	1.89	1.25
Age	0.95*	0.96	0.98	0.97
Bachelor’s degree	4.80**	1.72	2.75	2.15*
White	3.31*	1.14	2.41	1.41
Married	2.11	1.78	0.86	1.38

*p < 0.05; **p < 0.01; ***p < 0.001.

Table 7 includes the results of models comparing participants with high vs. low-to-moderate ACEs. PAEs were similarly associated with lower odds of depression in participants with high vs. lower ACEs. However, PAEs were only associated with lower odds of anxiety among those with high ACEs (0.82, *p* < 0.05). PCEs were not associated with anxiety or depression in either ACEs group.

**Table 7:** Adjusted odds ratios of PAEs on mental health in subsamples of high vs. low/moderate ACEs.

	Depression		Anxiety	
	High ACEs (≥4) (*n* = 152)	Low-to-moderate ACEs (<4) (*n* = 283)	High ACEs (≥4) (*n* = 152)	Low-to-moderate ACEs (<4) (*n* = 283)
PAEs	0.76**	0.76***	0.82*	0.88
PCEs	1.07	1.02	1.00	0.94
Female	0.56	2.81**	0.75	3.58**
Age	0.93**	0.98	0.96*	1.00
Bachelor’s Degree	6.65***	1.01	4.72**	0.97
White	1.43	2.12*	0.86	3.39*
Married	2.48	1.22	2.20	0.60

*p < 0.05; **p < 0.01; ***p < 0.001.

### 3.4. Aim 4: the association of PAEs and adult mental health among young vs. middle-aged adults

Young adults (<35 years) had 72% higher odds of having moderate-to-severe depression as compared to adults 35 years and older, but this relationship was not significant for anxiety. When the sample was split by age (see Table 8), PAEs were associated with a 33% decrease in odds for moderate-to-severe depression among young adults and a 20% decrease in odds for moderate-to-severe depression among adults ≥35 years. In anxiety models, PAEs were associated with 24% lower odds for moderate-to-severe anxiety among young adults, but the relationship was not significant among middle/older adults.

**Table 8:** Adjusted odds ratios of adult and childhood experiences on mental health, comparing young vs. middle/older adults.

	Depression		Anxiety	
	<35 years (*n* = 210)	≥35 years (*n* = 225)	<35 years (*n* = 210)	≥35 years (*n* = 225)
PAEs	0.67***	0.80**	0.76**	0.92
ACEs	1.72***	1.67***	1.80***	1.56***
PCEs	1.27**	1.05	1.14	0.99
Female	1.37	1.23	1.53	1.45
Bachelor’s degree	2.74*	1.94	2.53*	2.06
White	1.53	2.00	1.11	3.08*
Married	2.10	1.62	1.34	1.05

*p < 0.05; **p < 0.01; ***p < 0.001.

## 4. Discussion

The results partially substantiated the four hypotheses. Aligned with hypothesis 1, PCEs were predictive of positive experiences in adulthood. However, ACEs were not associated with PAEs. Supporting hypothesis 2, PAEs predicted less anxiety and depression in adulthood, regardless of their reported levels of PCEs and ACEs. PAEs were particularly protective against moderate-to-severe depression and anxiety among those with low PCEs or high ACEs (hypothesis 3). Finally, supporting hypothesis 4, younger adults (<35 years) appeared to derive more benefits from PAEs compared to adults 35 years and older.

The measures for both PCEs and PAEs consist of items evaluating connection to self, family, friends, and community. Extensive research has demonstrated the importance of connection, the antithesis of loneliness in both physical and mental health for children and adults (36, 37). The observed association between PCEs and PAEs may suggest that connections fostered in childhood may carry over into adulthood or improve adults’ ability to create and maintain similar connections and support.

Although we hypothesized that those with higher ACEs would have fewer PAEs, this was not supported. Additionally, among both those with high and lower-to-moderate ACE scores, PAEs were associated with reduced odds of depression. This suggests that the presence of adversity in childhood does not negate the ability for positive connection in adulthood and that adult social connection and support are more important for adult mental health than the experience of childhood adversity. This is hopeful, as many studies have examined ACEs from a deficit model that focuses on the long-term negative consequences of childhood adversity. While it is important to consider the challenges those who experience adversity have and will face, focusing on their capacity to have positive social connections and experiences in adulthood is more holistic and supports the growth of the individual.

Prior work has demonstrated that PCEs are more strongly associated with adult mental health than ACEs (2, 3). We observed a similar association in this study with respect to PAEs. Although preventing adversity in childhood is the goal, many people will experience adversity and these findings provide encouragement that the experience of adversity does not doom one to negative outcomes. PAEs may serve as an opportunity to turn the tide for those who have experienced childhood adversity and/or low levels of support or connection in childhood (PCEs). Fostering connection and support through positive adult experiences has the potential to positively influence mental health even in the presence of childhood adversity or low PCEs. We note that in the current study, PCEs were not directly associated with either anxiety or depression and in some models they were associated with higher odds for depression. These results are not consistent with prior research (2, 3). However, the primary message may be that PAEs appear to mediate the relationship between PCEs and adult mental health. Importantly, Bethell and colleagues found that PCEs were still associated with mental health even when controlling for a single-item measure of adult social and emotional support and relational health (2). Another explanation may include that as the data were collected during the COVID-19 pandemic, the import of earlier traumatic experiences became greater in a stressful time. In such cases, current positive experiences such as PAEs or healthy households were more important than prior positive experiences (38, 39). In models where PCEs were associated with higher odds for depression, this is likely explained by the strong correlation between PCEs and PAEs. It is possible that the findings may have been due to overcontrolling positive experiences in childhood and adulthood, though tests of multicollinearity did not indicate a problem. Regardless, these results may be best interpreted examining the indirect or mediating effect of PAEs on therelationship between PCEs and mental health.

Among the subgroup of respondents with a lower number of PCEs, higher PAEs were associated with decreased anxiety and depression. This may suggest that even in the presence of lower connection and support in childhood (as measured by lower PCEs) people can still establish positive adult experiences and social connections which are associated with better mental health outcomes. However, it is unclear whether PAEs result in improved mental health or if those with better mental health are more able to establish and maintain social connections (PAEs). Further, these associations likely function in a cyclical fashion whereas positive social connection improves mental health and good mental health strengthens social connections. Longitudinal research is needed to disentangle these relationships.

Early adulthood is a period of life characterized by significant transitions as young adults choose educational trajectories, lay the groundwork for occupational endeavors, and begin to establish family relationships outside their family of origin. As such, young adults may experience periods of intense stress associated with this “age of instability” (40). Further, emerging adulthood may be accompanied by increased struggles with mental health (41) and well-being perhaps complicated by instability in life situation and by the reduced social roles and obligations of this life stage (40). The instability of this period alone may result in inadequate social support for many young adults (42). This study observed that PAEs were associated with lower odds of depression and anxiety in younger adults (<35 years), as compared to older adults. While social support and connection are important for all humans, this finding supports the elevated value of adequate social connection and support, especially for younger adults.

It is noteworthy that participants with above the median PAEs were more likely to be married, report their race as White, have a Bachelor’s degree or higher, and less likely to have an income below $40,000/year. Given that the data were cross-sectional, it is difficult to ascertain whether PAEs contribute to stable marital relationships, higher income, and education or if the lack of a marital relationship, higher income, and/or higher education is prohibitive to the PAEs measured. Some of the PAE items may be linked with these demographic characteristics. For example, those with a Bachelor’s degree may be more likely to have a meaningful job, while those who have more money may have feel like they can engage in more fun activities, and being in a stable romantic relationship provides a built-in partnership with somebody who helps them make decisions about their life. It is also possible that some of the demographic differences between those with higher versus lower PAEs may have been due to the cultural appropriateness of the PAE questions themselves, and further research in diverse samples using the PAEs scale is paramount.

### 4.1. Implications for practice

Research has established that social connection can be an important risk or protective factor for health outcomes (37). As such, programs and policies that make it easier for people to develop close connections are important in reducing risk and increasing protection and ultimately improving health outcomes. Strengthening these connections through intervention can occur at each socioecological level. For example, at the community level, community mobilization efforts that bring community members together to address challenges and increase connection include Healthy Places by Design,^1^ the Blue Zones Project,^2^ and Communities That Care.^3^ Community-based programming that has a theoretical basis, offers social activity and support in a group format, and fosters active participation has been shown effective at reducing social isolation among seniors (43). At the family level, programming that works to improve family communication, resolve conflict, and improve cohesiveness includes Strengthening Families^4^ for all youth and parents and Functional Family Therapy^5^ for at-risk youth and parents. Finally, at the individual/peer level, interventions include befriending (introducing clients to others), mentoring (working with clients short-term to provide skills and abilities), and gatekeeping (connecting vulnerable clients with appropriate services) (44).

### 4.2. Limitations and strengths

Some study limitations are important to discuss. First, the data for this study were cross-sectional. As such, causality cannot be inferred. Longitudinal data are needed to better understand the relationships between PAEs and mental health and to examine potential reporter biases in reporting more positive experiences in childhood and currently. Second, data were collected in the fall of 2021 during the COVID-19 pandemic. As such, some responses and results may be affected by the pandemic and resulting trauma that some individuals and groups may have been experiencing. In particular, the relationship between PCEs and health in the current study is not consistent with prior studies using similar populations and methods (3, 45), and these inconsistencies may be due to underlying differences in daily life and mental health due to the COVID-19 pandemic. As such, it will be important to collect data after the pandemic to examine if relationships have changed, particularly as it relates to PAEs and health. Third, PAEs do not examine all possible positive adult experiences. Rather, they provide a snapshot of some key current cumulative positive experiences based on existing research relating to resilience and wellbeing in adulthood. Each experience was weighted equally although some experiences may have a greater positive impact on life and the quality of experience may vary from person to person. Participants had an average of 12.5 out of 15 PAEs and nearly 11 out of 13 PCEs, indicating a high degree of positivity. The high number of PCEs is consistent with prior studies (35), and future studies among diverse samples will demonstrate whether high positivity in adulthood is normative as well. Fourth, in future research researchers should consider using a Likert scale for response options rather than a binary (yes/no) response. This may help add more variability in responses and number of PAEs. As the PAEs scale is utilized in various samples, a next research step may be to examine different PAE conceptual groups (e.g., safety, emotional support, connection with community, and so forth) and their respective impact on health and wellbeing. Fifth, while we measured some forms of childhood adversity using an ACEs measure, we did not measure stressful life events in adulthood. Including adult stressful life events in future studies is important to understanding the degree to which PAEs may help prevent stressful life events, and when these events do occur, mitigating their effects on health and wellbeing. Finally, our sample was largely white and highly educated and the relationships observed may not be representative of low-income or other marginalized communities. Prior work on PCEs in a low-income sample showed similar results to more advantaged samples (35), therefore we would expect that PAEs will also be important in more disadvantaged groups. Items in the PAEs scale were derived from PCE scales that have been used in diverse samples (35). Nevertheless, additional work in a variety of settings and cultures is warranted and the PAEs items should be tested in other demographic groups to ensure cultural relevance.

Despite these limitations, the current study provides some insights as to how positive experiences may affect mental health even when ACEs were high and/or PCEs were low. Additionally, the new PAEs scale provides a measure of cumulative positive experiences in adulthood that had previously not been available for research. Continued refinement of the scale in other populations will be important to better examine how potential turning points (5) can help improve adult mental health even in the presence of adversity.

## Data availability statement

The datasets presented in this article are not readily available because the raw data may be made available upon request pending ethics committee approval. Requests to access the datasets should be directed to AC, ali_crandall@byu.edu.

## Ethics statement

The studies involving human participants were reviewed and approved by Brigham Young University Institutional Review Board. The patients/participants provided their written informed consent to participate in this study.

## Author contributions

AC, BM, and CH conceived of the study. AC oversaw the development of the PAEs scale, study design, data collection, conducted analyses, wrote the methods and results sections, and oversaw writing for the manuscript. BM, MB, HR, AP, and CH wrote the background and discussion. All authors assisted in the development of the PAEs scale and edited and reviewed the final manuscript.

## Conflict of interest

The authors declare that the research was conducted in the absence of any commercial or financial relationships that could be construed as a potential conflict of interest.

## Publisher’s note

All claims expressed in this article are solely those of the authors and do not necessarily represent those of their affiliated organizations, or those of the publisher, the editors and the reviewers. Any product that may be evaluated in this article, or claim that may be made by its manufacturer, is not guaranteed or endorsed by the publisher.

## Footnotes

^1^ http://healthyplacesbydesign.org

^2^ http://bluezones.com

^3^ http://communitiesthatcare.net

^4^ http://extension.iastate.edu/sfp10-14

^5^ http://www.fftinc.com
